# The feasibility and efficacy of implementing a focused cardiac ultrasound course into a medical school curriculum

**DOI:** 10.1186/s12909-017-0928-x

**Published:** 2017-05-30

**Authors:** Sergio L. Kobal, Yotam Lior, Alon Ben-Sasson, Noah Liel-Cohen, Ori Galante, Lior Fuchs

**Affiliations:** 1 Cardiology Department, Soroka University Medical Center, Beer-Sheva, Israel; 2 Clinical Research Center, Soroka University Medical Center, Beer-Sheva, Israel; 3 Medical Intensive Care Unit, all at Soroka University Medical Center and The Faculty of Health Sciences, Ben-Gurion University of the Negev, Beer-Sheva, Israel

## Abstract

**Background:**

Teaching cardiac ultrasound to medical students in a brief course is a challenge. We aimed to evaluate the feasibility of teaching large groups of medical students the acquisition and interpretation of cardiac ultrasound images using a pocket ultrasound device (PUD) in a short, specially designed course.

**Methods:**

Thirty-one medical students in their first clinical year participated in the study. All were novices in the use of cardiac ultrasound. The training consisted of 4 hours of frontal lectures and 4 hours of hands-on training. Students were encouraged to use PUD for individual practice. Finally, the students’ proficiency in the acquisition of ultrasound images and their ability to recognize normal and pathological states were evaluated.

**Results:**

Sixteen of 27 (59%) students were able to demonstrate all main ultrasound views (parasternal, apical, and subcostal views) in a six-minute test. The most obtainable view was the parasternal long-axis view (89%) and the least obtainable was the subcostal view (58%). Ninety-seven percent of students correctly differentiated normal from severely reduced left ventricular function, 100% correctly differentiated a normal right ventricle from a severely hypokinetic one, 100% correctly differentiated a normal mitral valve from a rheumatic one, and 88% correctly differentiated a normal aortic valve from a calcified one, while 95% of them correctly identified the presence of pericardial effusion.

**Conclusions:**

Training of medical students in cardiac ultrasound during the first clinical year using a short, focused course is feasible and enables students with modest ability to acquire the main transthoracic ultrasound views and gain proficiency in the diagnosis of a limited number of cardiac pathologies.

**Electronic supplementary material:**

The online version of this article (doi:10.1186/s12909-017-0928-x) contains supplementary material, which is available to authorized users.

## Background

The diagnosis of cardiac disease is based on medical history, physical examination, and complementary studies. The cardiovascular physical exam, based mainly on cardiac auscultation, can be performed during the first patient-doctor encounter. However, its diagnostic accuracy is suboptimal [1–8]. Some cardiovascular pathologies are difficult to identify by means of physical examination due to imperceptible or barely detectable clinical manifestations [4, 6, 9–12], with no connection to physicians’ skills. In order to improve their diagnostic capability, physicians make use of complementary diagnostic imaging techniques. Ultrasound offers precise anatomical and functional information on the cardiovascular system and is the most commonly used technique for diagnosing cardiovascular diseases [2, 6, 10, 13, 14].

Decara et al. showed that the utilization of hand-held ultrasound devices significantly augmented the accuracy of medical students’ cardiac diagnoses at the bedside compared to traditional physical examination [3]. However, the practicability of echocardiography is limited due to the cost of the device, its cumbersome size, and the lack of availability of expert personnel to perform and interpret studies.

New, small, user-friendly ultrasound equipment has been in use for the last decade. Recently, the pocket ultrasound device (PUD), with high imaging resolution, has become commercially available [14]. The PUD has the same characteristics as the traditional stethoscope routinely used by physicians in their physical examinations for the last 150 years: it is portable, free of adverse effects, and has no contraindications. The PUD allows for a quick and accurate observation of a patient’s internal structures as well as their function, and thus enhances the diagnostic potential of the “auscultation-assisted” physical examination [1–5, 8, 11, 14–18].

Acquiring ultrasound skills in medical school is vitally important, but teaching these skills presents challenges. Recently, a consensus of 34 experts in the field of ultrasound in medical education established recommended ultrasound training milestones that all medical students should reach before they graduate [19].

The main limitations of ultrasound-assisted physical examination are related to time of training until proficiency is achieved in obtaining echo views and in the ability to identify cardiac pathologies. Teaching the acquisition of cardiac ultrasound imaging is based mainly on hands-on practice in a one-on-one fashion, under the direct supervision of highly qualified instructors. This makes the teaching of large numbers of medical personnel unfeasible, primarily due to the limited number of instructors and teaching costs.

## Methods

Devising a time-efficient and effective method of teaching cardiac ultrasound to larger groups is challenging, but could facilitate the incorporation of this important diagnostic modality into medical school curriculums.

Our study’s objectives were to assess the feasibility and efficacy of having novice medical students perform and read basic cardiac ultrasound studies during their first clinical rotation after participating in a newly designed, brief (eight-hour), focused cardiac ultrasound course using a PUD. Specifically, we aimed to validate this teaching method by testing whether students could achieve standard echocardiographic views and analyze basic transthoracic cardiac ultrasound images and basic cardiac pathologies.

### Study population

A total of 31 medical student volunteers in their first clinical year (fourth year of a six-year medical school program) were enrolled in the study. The course took place during the first 2 weeks of an eight-week internal medicine rotation. The importance of the course was conveyed to the class and the schedule was presented a month before the initiation of the rotation. Next, the students were notified simultaneously about the course and all the students agreed to participate in it. The students all signed an informed consent form. None of the students had any prior experience with the use of ultrasound, so pretesting did not appear to be necessary. The results of students’ performance in this study were not available to medical school faculty and therefore did not influence their evaluations.

### The intervention

The course consisted of frontal lectures and hands-on training sessions. The course objectives were to teach students how to obtain standard cardiac ultrasound images from standard transthoracic views and how to recognize normal cardiac ultrasound anatomy as well as pathological states of the left ventricle, the right ventricle, the mitral and aortic valves, the inferior vena cava, and the pericardium.

Students’ training consisted of 4 hours of frontal lectures and an additional 4 hours of hands-on practice under the direct supervision of cardiologists and echocardiographic sonographers. The eight-hour course was incorporated into the eight-week internal medicine rotation in place of lectures that students could view independently in a pool of lectures that is available to them as e-Learning material. During their eight-week rotation, following the course, students were encouraged to use PUD autonomously (without supervision), practicing image acquisition on healthy volunteers and hospitalized patients. Students were asked to record acquired images for later revision with a tutor for feedback and skill improvement. This post-course self-training was not obligatory.

### Lectures

The 4 hours of lectures were divided into two weekly sessions of 2 hours each. The subjects covered in the lectures were the following: basic ultrasound physics, principles of two-dimensional ultrasound imaging, the Doppler effect, and cardiac ultrasound anatomy from parasternal, apical, and subcostal views. Previous clinical echocardiographic cases were used to teach students pattern recognition of normal and abnormal LV and RV function, identification of aortic valve structure (normal, bicuspid, and degenerative calcified), normal mitral valve versus mitral valve with rheumatic damage, presence of pericardial effusion, and dilated versus normal IVC. Color Doppler imaging was used to teach students to identify aortic and mitral regurgitation of any degree.

### Hands-on practice

During the same week the lectures were given, students had a total of 4 hours of guided hands-on training (divided into two sections of 2 hours each). Students practiced image acquisition utilizing standard ultrasound devices (Vivid 6 and Vivid I, General Electric). These devices optimized image acquisition for teaching purposes by providing a larger screen and better image quality than the pocket devices. Training sessions were led by cardiologists or echocardiographic sonographers with teaching experience on healthy student volunteers who served as models. Training was performed in small groups (up to four students) and focused on the acquisition of standard cardiac ultrasound images.

**Fig. 1:**
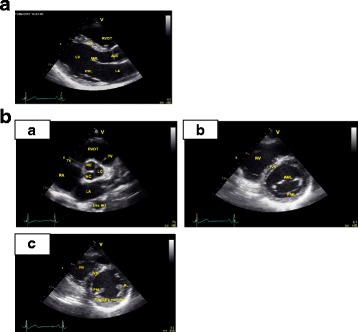
**a** Parasternal long axis view and main anatomic points. **b** Parasternal short axis view at three levels. *a* Aortic level. *b* Mitral level. *c* Mild papillary level

**Fig. 2:**
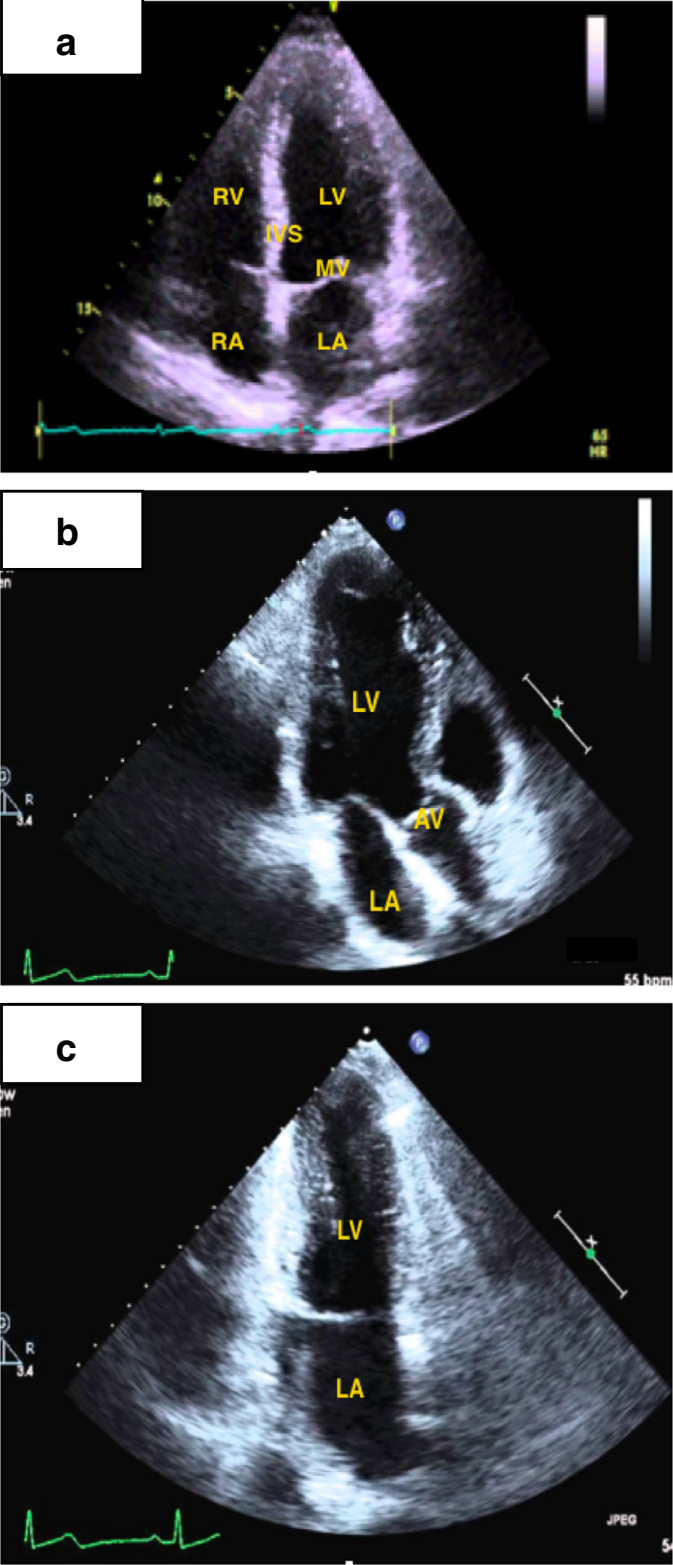
Apical 4, 3 and 2 chamber views. **a** Aplical 4 chamber view. **b** Aplical 3 chamber view. **c **Aplical 2 chamber view

**Fig. 3:**
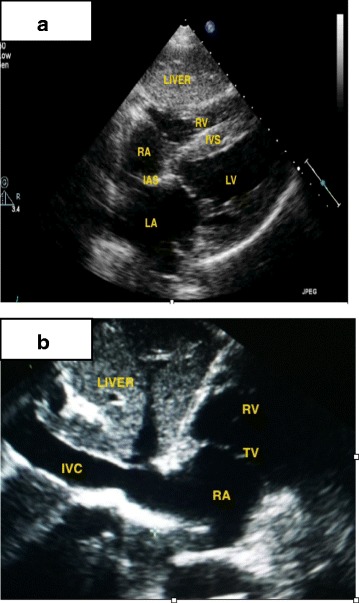
Subcostal views. **a** Subcostal long axis view. **b** Subcostal IVC view

The practiced views included the parasternal long-axis view, the parasternal short-axis view (three levels: aortic, mitral, and midpapillary), the apical four-, two-, and three-chamber views (4-ch, 2-ch, 3-ch respectively), and the subcostal view. Students were taught to move the transducer with gentle maneuvers and to perform three main motions: alignment, rotation, and tilt, in order to produce the optimal image in each of the ultrasound windows. Students were also instructed to use general 2D gain and image depth controls for better ultrasound images. Figures 1, 2 and 3 show details of the anatomical points that the students had to obtain in each of the ultrasound views.

### Individual practice using the pocket ultrasound device

Students were encouraged to practice image acquisition during the eight-week clinical rotation. This PUD practice was completely voluntary and no number of required practice hours was defined. Every five students were provided with a PUD (Vscan from General Electric) for individual practice. The easy-to-use device is shaped like a small flip-phone and has an attached 2.5 MHz cardiac transducer. It has a 3.5-in. display screen and weighs 390 g. With two separate control settings (depth and gain), the Vscan produces a black-and-white real-time two-dimensional ultrasound image as well as a color-coded blood flow image. Images can be frozen and simple linear measurements can be performed. All images produced with the device can be saved on the system’s memory card for later viewing and feedback.

Practice was neither predefined nor mandatory. Students performed cardiac ultrasound studies on one another as well as on their patients as part of the physical examination. We therefore advised our students to inform the patients they examined that the ultrasound study they performed did not in any way replace a comprehensive echocardiographic study, and that no diagnosis or clinical decision would be made based on it.

### Post-intervention assessment

At the end of the eight-week clinical rotation, enrolled students underwent two exams. The first was designed to assess hands-on cardiac ultrasound image acquisition of the main transthoracic views (the six-minute exam, see below), while the second aimed to validate their abilities to recognize different cardiac ultrasound views, particularly their ability to differentiate normal from pathologic ventricular, valvular, IVC, and pericardial findings (ultrasound image interpretation exam).

### The six-minute exam

The goal of this exam was to assess students’ ability to produce transthoracic cardiac ultrasound images. Each student had 6 minutes to obtain the main echocardiographic views in a predetermined order using a PUD: PLAV (Fig. 1a), PSAV (at the aortic, mitral, and mid-papillary levels; Fig. 1b), apical views (4-ch, 2-ch, 3-ch; Fig. 2) and subcostal views (standard and focused on IVC; Fig. 3).

During the exam, the supervising physician used a checklist of cardiac anatomical points that each student was required to identify in each one of the main transthoracic ultrasound views and a final score was obtained according to the student’s performance (Additional file 1: Appendix A). The views were obtained on healthy models who were pre-screened and had good ultrasound windows.

By the end of the six-minute period, the exam ended, regardless of the number of windows acquired and the students were asked to report their personal self-training time during the rotation in hours.

### Ultrasound image interpretation exam

The exam consisted of 23 questions, each with one echo clip. The students were evaluated for their ability to identify the main cardiac ultrasound views and recognize normal and abnormal LV and RV function, the anatomy of the aortic and mitral valves, IVC size and respiratory variation, valvular dysfunction, and the presence of pericardial effusion.

### Statistical analysis

The data collected was documented using summary tables. Continuous variables with normal distribution were presented as mean and standard deviation with 95% confidence interval when appropriate. Ordinary variables or continuous variables with non-normal distribution were presented as median with an interquartile range (IQR). Categorical variables were presented as counts and percentages of the total.

Continuous variables were analyzed using student t-test. Non-parametric procedures (Mann–Whitney) were used if parametric assumptions could not be satisfied even after data transformation attempts. Parametric model assumptions were assessed using Normal-plot or the Shapiro-Wilks test for verification of normality and Levene’s test for verification of homogeneity of variances.

Categorical variables were tested using Pearson’s χ2 test for contingency tables or Fisher Exact test when appropriate. Correlations between variables were tested using Pearson or Spearman tests, depending on variable distribution.

All statistical tests and/or confidence intervals, as appropriate, were performed at α = 0.05 (two-sided) and presented with their 95% confidence interval when appropriate.

## Results

All 31 students completed the eight-hour course, as required by the study protocol. All students but one could visualize the parasternal long-axis view (PLAV), all visualized at least one level (aortic, mitral, mid-papillary) of the parasternal short-axis view (PSAV), all visualized at least one of the three apical views (four-, three- and two-chamber views) and 89% visualized at least one anatomical view of the subcostal view (Table 1).

**Table 1:** The six-minute exam of ability to obtain correct ultrasound images

Ultrasound views and defined anatomical points			Score
Parasternal Long-Axis View			88.89 ± 23.34
	Correct alignment		20/27 (74.1%)
	Endocardial demarcation		24/27 (88.9%)
	Mitral valve visualization		26/27 (96.3%)
	Aortic valve visualization		26/27 (96.3%)
Parasternal Short-Axis View			78.24 ± 24.41
	Aortic level demonstration		69.4 ± 37.6
		Aorta visualization	22/27 (81.5%)
		Tricuspid valve visualization	21/27 (77.8%)
		Pulmonic valve visualization	18/27 (66.7%)
		Interatrial septum visualization	14/27 (51.9%)
	Mitral level demonstration		85.2 ± 30.43
		Complete left ventricle visualization	21/27 (77.8%)
		Mitral valve visualization	25/27 (92.6%)
	Midpapillary level demonstration		88.9 ± 32
		Complete left ventricle visualization	24/27 (88.9%)
		Papillary muscle visualization	24/27 (88.9%)
Apical View			62.96 ± 24.29
	Four-Chamber View		59.3 ± 34.4
		Left ventricle visualization	11/27 (40.7%)
		Right ventricle visualization	13/27 (48.1%)
		Mitral valve visualization	24/27 (88.9%)
		Tricuspid valve visualization	22/27 (81.5%)
		Atrium visualization	10/27 (37%)
	Two-Chamber View		61.7 ± 38.9
		Left ventricle visualization	14/27 (51.9%)
		Mitral valve visualization	23/27 (85.2%)
		Left atrium visualization	13/27 (48.1%)
	Three-Chamber View		67.9 ± 41.8
		Left ventricle visualization	15/27 (55.6%)
		Mitral valve visualization	21/27 (77.8%)
		Aortic valve visualization	19/27 (70.4%)
Subcostal View			57.78 ± 34.34
		Right ventricle visualization	18/27 (66.7%)
		Pericardial visualization	20/27 (74.1%)
		Interatrial septum visualization	11/27 (40.7%)
		Inferior vena cava visualization	16/27 (59.3%)
		Inferior vena cava respiratory variation visualization	13/27 (48.1%)

The average grade on the cardiac ultrasound view recognition test was 73%. Thirty students (97%) were able to correctly identify normal LV function and LV dysfunction. All 31 students differentiated normal mitral valve from rheumatic mitral valve injury and 95% of the students diagnosed the presence of pericardial effusion.

The average PUD individual practice time (which was not obligatory) was 3.3 ± 2.7 h per student. The maximum individual practice time was 10 h (reported by one student). Four students reported no individual practice at all.

### The six-minute exam

Twenty-seven of 31 (87%) students performed the exam. All participants were able to obtain at least one anatomical view of the eight transthoracic views within the 6 minutes allowed. The most obtainable view (the view that the largest number of students could achieve) was the PLAV, with a median rate of 89% (SD 23%), while the least obtainable view was the subcostal view, with a median rate of 58% (SD 34%).

### Parasternal views

Twenty-six of 27 students (96%) obtained at least one image with one of the four anatomical points required for the PLAV view (correct alignment, total endocardial demarcation of LV, and visualization of mitral leaflets or aortic valve cusps). Although seven students (26%) could not achieve the correct alignment for PLAV anatomical points, endocardial demarcation and mitral and aortic valves were visualized by most students (89%, 96%, and 95% respectively; Table 1).

All students were able to obtain at least one image of the eight anatomical points defined for the PSAV view at the aortic level (aortic, tricuspid, and pulmonic valves, interatrial septum), mitral level (complete LV endocardial demarcation, mitral valve), and midpapillary level (complete LV endocardial demarcation, papillary muscles). Students performed better in scanning the midpapillary than the mitral or the aortic level (average grades of 89%, 85%, and 78% respectively; Table 1).

### Apical views

All of the students were able to obtain at least one of the three apical views, namely 4-ch, 2-ch, and 3-ch. Eighteen students (67%) obtained all apical views. Six (22%) students obtained two out of the three apical views, and three (11%) students were able to obtain only one of the three apical views. The most obtainable apical view was 4-ch (89%) and the least obtainable was 3-ch (77%). Table 1 shows that students had less success in imaging entire ventricles (up to 56% for the LV and up to 48% for the RV) than for imaging the valves (up to 89% for the mitral and 70% for the aortic valve) from the apex.

### Subcostal view

The subcostal view comprised the last part of the six-minute exam and the students were required to show two views: the long-axis four-chamber standard view and the tilted view of the IVC. Twenty-four of 27 students (89%) were able to produce at least one anatomical view of five predetermined anatomical points for this view (Appendage A). Imaging of the IVC was achieved by 16 of 27 students (59%). The rest of the results for this view are summarized in Table 1.

### Ultrasound image interpretation exam

This exam was divided into different topics: cardiac view recognition, ventricular function identification, valvular abnormality identification, pericardial abnormality recognition, and IVC recognition.

### Cardiac view recognition

All 31 students took the exam. The average grade on cardiac ultrasound view recognition was 73 ± 30. Table 2 shows the students’ scores for the interpretation of cardiac ultrasound images.

**Table 2:** Ultrasound image interpretation exam

Cardiac view recognition		
	Parasternal long-axis view	26/31 (83.8%)
	Parasternal short-axis view – Base	19/31 (61.3%)
	Parasternal short-axis view – Mid-ventricle	19/31 (61.3%)
	Parasternal short-axis view – Apex	20/31 (64.5%)
	Apical 4-chamber	29/31 (93.5%)
	Apical 2-chamber	22/31 (70.9%)
	Apical 3-chamber	19/31 (61.3%)
	Subcostal	26/31 (83.9%)
Ventricular Function		
	Left ventricle – Normal function	30/31 (96.8%)
	Left ventricle – Dysfunction	30/31 (96.8%)
	Left ventricle – Hyperdynamic contractility	28/31 (90.3%)
	Left ventricle – Regional wall motion contractility	25/31 (80.6%)
	Right ventricle – Normal function	31/31 (100%)
Mitral and Aortic Valves		
	Mitral valve – Normal structure	31/31 (100%)
	Mitral valve – Rheumatic injury	31/31 (100%)
	Mitral valve – Regurgitation	25/31 (80.6%)
	Aortic valve – Normal structure	10/11 (90.9%)
	Aortic valve – Bicuspid valve	8/31 (25.8%)
	Aortic valve – Calcified	22/31 (71%)
Other		
	Dilated cardiomyopathy	25/31 (80.6%)
	Pericardial effusion	19/20 (95%)
	Dilated inferior vena cava	16/31 (51.6%)

### Left and right ventricular function

Thirty students (97%) were able to correctly identify normal LV function and LV dysfunction. Twenty-eight of 31 (90%) students were able to recognize hyperdynamic LV function. All the students were able to differentiate normal from severely hypokinetic RV.

### Mitral and aortic valves

All the students differentiated normal mitral valve from rheumatic mitral valve injury. Twenty-two students (71%) were able to recognize a calcified aortic valve. Bicuspid aortic valve was diagnosed by 8 of 31 (25%) students.

Twenty-five students (81%) were able to recognize a jet of mitral regurgitation and 17 students (54%) identified a regurgitant jet of aortic regurgitation by color Doppler.

### Pericardium and IVC

Ninety-five percent of the students diagnosed the presence of pericardial effusion from the subcostal view. Dilated IVC was recognized by 16 students (52%).

## Discussion

We designed a brief cardiac ultrasound course for medical students during their first clinical year with the aim of providing them with tools for expanding the physical examination. This condensed course combines 4 hours of lectures for the entire group, 4 hours of small-group hands-on sessions, and additional elective individual practice time with a PUD.

This is a “proof of concept” study that shows that teaching a large group of students to conduct basic cardiac ultrasound exams in a relatively short period of time is feasible. Students were also able to accurately differentiate normal from severely decreased LV and RV function, normal mitral valves from rheumatic mitral valve injury, and normal aortic valves from calcified aortic valves, and to identify pericardial effusion correctly.

Numerous studies have demonstrated the feasibility of teaching medical students, residents, and novice non-cardiologists the performance and interpretation of cardiac ultrasound [1, 3, 4, 8, 11, 12, 16–18, 20–27]. Our results are similar to those of some of the previously published studies (by our group and others), which showed that novice personnel and medical students can, after brief training, accurately differentiate severe LV dysfunction from normal function, diagnose rheumatic injury of the mitral valve [27], and recognize the presence of pericardial effusion after just a few hours of training [11, 12, 22, 28]. However, none of these previous studies were conducted on a large group of medical students during their first clinical year, objectively assessing their hands-on performance as well as their ability to interpret basic pathologies.

Panoulas and colleagues trained only five final-year medical students after their cardiology rotation and showed improved clinical diagnosis [21]. Andersen and colleagues trained 30 fifth-year medical students, but in this study the intensity (teacher-student ratio) of hands-on teaching was not specified and the study suffered from selection bias, as the assessment of scanning quality was dependent on students’ log books, where students chose their best clips and were not evaluated in an objective test as they were in our study [11]. Stokke and colleagues briefly trained (in a four-hour course) 21 medical students using PUD, but compared their findings to auscultation and did not present objective scanning quality assessment [29].

We have objectively measured students’ performance and showed that a large class can become proficient in conducting and assessing basic cardiac ultrasound clips by participating in relatively short hands-on training in groups of four. Our students did only fairly well in terms of their ability to acquire all cardiac images. Sixteen of 27 (59%) students were able to demonstrate all the views (parasternal, apical, and subcostal) within 6 minutes. But most students obtained at least one transthoracic echocardiographic view, while the most obtainable view was the parasternal long-axis view (89%) and the least obtainable was the subcostal view (58%). It is possible that the six-minute test was too short to allow novice operators to obtain all main cardiac views. This notion is supported by the fact that the last view to be obtained in the exam (the subcostal view) was also the least obtainable. A longer exam would probably have improved students’ scores.

Another novel intervention was the incorporation of non-obligatory individual PUD practice time during the eight-week clinical rotation. This teaching modality may have contributed to the students’ performance as measured by the six-minute exam. However, we could not assess the correlation between the number of individual practice hours and final exam scores due to inadequate self-reporting by the students.

Strzecka and colleagues recently showed a learning curve effect among novice operators after a short course on PUD. Medical students detected major abnormalities with an acceptable diagnostic value that increased with the number of examinations performed [12]. Ruddox and colleagues found poor scanning performance when medical residents received only a two-hour training program (a one-hour practical demonstration followed by a one-hour hands-on training session) [22]. Students demonstrated poor ability to recognize impaired cardiac function, pericardial effusion, and valvular heart disease after this very brief training. Anderson and colleagues trained 30 fifth-year volunteer medical students who received a total of 9 hours (three sessions) of combined practical and theoretical training in the use and interpretation of ultrasound images. Students were encouraged to perform at least 75 PUD examinations prior to being tested [11]. Seventy-four percent of the students recorded acceptable cardiovascular organ imaging. In agreement with our results, students were better at interpreting cardiac images than acquiring them. Their diagnostic accuracy in correctly identifying reduced LV function, pericardial effusion, pleural effusion, lung comets, IVC diameter, and respiratory variation was 94% (CI 89.0–96.5). At present, the recommendations regarding a minimum number of studies (performance of at least 75 echocardiographic studies and interpretation of 150 complete studies under supervision) are indicated for sonographers and physicians who are learning comprehensive echocardiographic studies with premium echocardiographic devices. Due to inconsistent data that exists to date, there is no consensus on the minimum number of studies or hours of training needed to teach cardiac ultrasound as a diagnostic tool in medical schools.

A prolonged teaching program would be impractical when teaching novice medical personnel on a large scale. On the other hand, very short training could be insufficient to achieve the true benefit of ultrasound-assisted physical examination and comprise a source of mistakes and misdiagnoses. It seems that at least 8 hours of PUD training (according to our results and previously published data) may be sufficient for medical students to learn to accurately assess basic cardiac parameters by echocardiography, but insufficient to generate independent ultrasound cardiac imaging using pocket devices. The acquisition of the ability to use PUDs independently may be enhanced by more structured guidance for individual use of PUD during clinical rotations. We do believe that the “consolidation process” of bedside ultrasound studies is directly related to the availability of PUDs for student use. In our hospital, each internal medicine ward has one PUD device. In addition, in our course we used four PUDs provided by the cardiology department of the hospital affiliated with the medical school to the students for their free practice during the eight-week internal medicine rotation. The PUDs were available for each student every 4 days. In our estimation, this number of PUDs allowed our students to practice ultrasound image acquisition at an acceptable frequency.

Practicing by utilizing PUD in relevant medical school clinical rotations will improve students’ ability to perform focused cardiac ultrasound examinations. We believe that incorporating the six-minute hands-on exam and the pathological clip exam into the final rotation’s formal student assessment as well as requiring students in clinical rotations to submit logbooks of PUD images will further improve their bedside point-of-care ultrasound performance.

Our study has some limitations. Students did not accurately report self-training time, and thus provided only estimated data that limited our ability to reliably test the correlations between the amount of self-training and the final exam scores. Perhaps obligatory guided self-training sessions during the clinical rotation would have improved hands-on performance on the six-minute exam. In addition, we used young, healthy models, known to have good acoustic windows, for the six-minute hands-on test, and not actual medical patients.

The ultrasound image recognition exam made use of pathological pre-recorded clips, which were presented to the students on a screen instead of real patients with pathologies in real medical settings. No pretest was needed as none of the students had any prior experience with the use of ultrasound.

Teaching cardiac ultrasound to large groups of medical students is challenging. Incorporating ultrasound into anatomy and physiology curriculums in the preclinical years could improve students’ skills during the clinical years. Replacing highly qualified sonographers and cardiologists with medical students who have received formal instruction could provide an option for coping with the reality of a limited number of instructors being available for hands-on practice [25, 26]. More support of training in the wards may be possible once more physicians begin using this tool as part of regular practice during clinical rounds. The use of web-based learning modules as well as simulators (currently available for learning basic as well as advanced ultrasound) to complement practice sessions are options that we are assessing both to reduce costs and to make this type of course more efficient.

## Conclusions

Large-scale instruction of medical students in cardiac ultrasound during the first clinical years is feasible. Brief training in cardiac ultrasound that includes lectures, hands-on sessions, and individual practice allows medical students to gain proficiency in the diagnosis of a limited number of cardiac states and provides them with a modest ability to acquire transthoracic ultrasound views. Therefore, in order to modify traditional methods of physical examination and diagnosis by including ultrasound assessment as a complement of the physical examination, it seems appropriate to incorporate basic courses similar to the one described here into medical school curriculums.

## Additional file

Additional file 1: Appendix A. (DOCX 92 kb)

## Abbreviations

AL: Anterolateral
AML: Anterior mitral leaflet
AoV: Aortic valve
Des AO: Descending Aorta
IAS: Interatrial septum
IVC: Inferior vena cava
IVS: Interventricular septum
LA: Left atrium.
LC: Left coronary cusp
LV: Left ventricle
NC: Noncoronary cusp
PM: Posteromedial
PML: Posterior mitral leaflet
PV: Pulmonic valve
RA: Right atrium
RC: Right coronary cusp
RV: Right ventricle
RVOT: Right ventricle outflow tract
TV: Tricuspid valve
