# Supplementary material for: The feasibility and efficacy of implementing a focused cardiac ultrasound course into a medical school curriculum
Source: BMC Med Educ. 2017 May 30;17:94. doi: 10.1186/s12909-017-0928-x (PMC5450418; doi:10.1186/s12909-017-0928-x)
Supplement: Additional file 1: — Appendix A. (DOCX 92 kb) [file 12909_2017_928_MOESM1_ESM.docx]

**APPENDAGE A: 6-MINUTE EXAM**

**PARASTERNAL LONG-AXIS VIEW**

1 Correct alignment 0 Incorrect alignment

1 Total endocardial demarcation 0 Partial endocardial demarcation

1 Mitral valve visualization 0 Not visualized

1 Aortic valve visualization 0 Not visualized

**PARASTERNAL SHORT-AXIS VIEW BASE**

AORTA VISUALIZATION 1 YES 0 NO

TRICUSPID VALVE 1 YES 0 NO

PULMONIC VALVE 1 YES 0 NO

INTERATRIAL SEPTUM 1 YES 0 NO

**PARASTERNAL SHORT-AXIS VIEW MID-VENTRICLE**

COMPLETE LV VISUALIZATION 1 YES 0 NO

MITRAL VALVE VISUALIZATION 1 YES 0 NO

**PARASTERNAL SHORT-AXIS VIEW APEX**

COMPLETE LV VISUALIZATION 1 YES 0 NO

PAPILLARY MUSCLES VISUALIZATION 1 YES 0 NO

**APICAL 4-CHAMBER VIEW**

OPEN LV 1 YES 0 NO

RV VISUALIZATION 1 YES 0 NO

MITRAL VALVE ANATOMY 1 YES 0 NO

TRICUSPID ANATOMY 1 YES 0 NO

OPEN ATRIUM 1 YES 0 NO

**APICAL 2-CHAMBER VIEW**

OPEN LV 1 YES 0 NO

MITRAL VALVE ANATOMY 1 YES 0 NO

OPEN LA 1 YES 0 NO

**APICAL 3-CHAMBER VIEW**

OPEN LV 1 YES 0 NO

MITRAL VALVE ANATOMY 1 YES 0 NO

AORTIC VALVE ANATOMY 1 YES 0 NO

**SUBCOSTAL VIEW**

OPEN RV 1 YES 0 NO

PERICARDIAL DEMARCATION 1 YES 0 NO

INTERATRIAL SEPTAL VISUALIZATION 1 YES 0 NO

IVC VISUALIZTION 1 YES 0 NO

IVC RESPIRATORY VARIATION 1 YES 0 NO
